# Linking the relationship between dietary folic acid intake and risk of osteoporosis among middle‐aged and older people: A nationwide population‐based study

**DOI:** 10.1002/fsn3.4070

**Published:** 2024-03-05

**Authors:** Yuan‐Wei Zhang, Yan Hu, Si‐Cheng Wang, Zu‐Hao Li, Gui‐Quan Cai, Hao Shen, Shi‐Hao Sheng, Xiao Chen, Wei‐Zong Weng, Wen‐Cai Zhang, Yuan Chen, Jia‐Can Su

**Affiliations:** ^1^ Department of Orthopaedics Xinhua Hospital Affiliated to Shanghai JiaoTong University School of Medicine Shanghai China; ^2^ Institute of Translational Medicine Shanghai University Shanghai China; ^3^ Organoid Research Center Shanghai University Shanghai China; ^4^ National Center for Translational Medicine (Shanghai) SHU Branch Shanghai University Shanghai China; ^5^ Department of Orthopaedics Shanghai Zhongye Hospital Shanghai China; ^6^ Department of Orthopaedics The First Affiliated Hospital of Jinan University Guangzhou Guangdong China; ^7^ Department of Orthopaedics and Traumatology, Nanning Hospital of Traditional Chinese Medicine Guangxi University of Chinese Medicine Nanning Guangxi China

**Keywords:** folic acid, middle‐aged and older people, osteoporosis, population‐based study

## Abstract

Among middle‐aged and older people, balanced and nutritious diets are the foundation for maintaining bone health and preventing osteoporosis. This study is aimed at investigating the link between dietary folic acid intake and the risk of osteoporosis among middle‐aged and older people. A total of 20,686 people from the National Health and Nutritional Examination Survey (NHANES) 2007–2010 are screened and included, and 5312 people aged ≥45 years with integral data are ultimately enrolled in evaluation. Demographics and dietary intake‐related data are gathered and analyzed, and the odds ratio (OR) and 95% confidence interval (CI) of each tertile category of dietary folic acid intake and each unit increase in folic acid are assessed via multivariate logistic regression models. On this basis, the receiver operating characteristic (ROC) curve is used to identify the optimal cutoff value of dietary folic acid intake for indicating the risk of osteoporosis. Of 5312 people with a mean age of 62.4 ± 11.0 years old, a total of 513 people with osteoporosis are screened, and the dietary folic acid intake amount of the osteoporosis group is significantly lower than that of the non‐osteoporosis group (*p* < .001). The lowest tertile category is then used to act as a reference category, and a higher dietary folic acid intake amount is observed to be positively related to lower odds for risk of osteoporosis. This trend is also not changed in adjustments for combinations of different covariates (*p* all < .05). Based on this, a dietary folic acid intake of 475.5 μg/day is identified as an optimal cutoff value for revealing osteoporosis. Collectively, this nationwide population‐based study reveals that a higher daily dietary folic acid intake has potential protective effects on osteoporosis in middle‐aged and older people.

## Graphical abstract

This study is aimed at investigating the link between dietary folic acid intake and the risk of osteoporosis among middle‐aged and older people, and a total of 20,686 people from the National Health and Nutritional Examination Survey (NHANES) 2007–2010 were screened and included. The results indicate that a higher daily dietary folic acid intake has potential protective effects on osteoporosis in middle‐aged and older people.

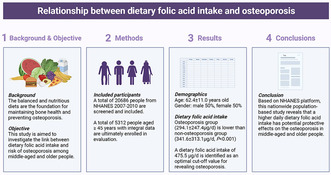

## 1 INTRODUCTION

Osteoporosis is the most universal systemic metabolic bone disease among middle‐aged and older people, which is characterized by persistent reduction of bone mass and degradation of bone microstructure, leading to enhanced bone fragility and the occurrence of fractures (Miller et al., 2022; Zhang, Cao, Li, Dai, Lu, Zhang, Bai, Chen, Zhang, et al., 2022; Zhou et al., 2023). Of note, osteoporotic fracture means a low‐energy or non‐violent fracture that occurs without apparent external force, which is also a major and non‐negligible complication of osteoporosis (Pinto et al., 2022; Wang et al., 2021). Currently, with the ceaseless progression of the global population aging, the number of patients with fractures caused by osteoporosis is as high as 8.9 million annually (Zhang, Song, et al., 2024), which means that there is one osteoporotic patient complicated with fractures every 3 s (Mills et al., 2022). Besides, it is expected that by 2050, China will also become the country with the highest incidence of osteoporotic fractures in Asia (Wang et al., 2021), which might not only affect the quality of life of middle‐aged and older people but also enhance the burden of the social medical service system to certain extents (Chen et al., 2021; Guo et al., 2023; Hu, Li, Zhang, et al., 2021; Hu, Li, Zhi, et al., 2021; Liu et al., 2022, 2023).

Additionally, a healthy, reasonable, balanced, and nutritious diet is the foundation for maintaining the bone and whole health of middle‐aged and older people (Farshbaf‐Khalili et al., 2023; Kheiridoost et al., 2022; Li et al., 2024), and the prevention and treatment of osteoporosis also requires cooperative management from multiple perspectives (Warensjö et al., 2011; Zhang, Cao, Li, Dai, Lu, Zhang, Bai, Chen, Shi, et al., 2022). In addition to actively seeking medical treatment, it is necessary to pay attention to rational diets in daily life to supplement the necessary nutrients for the bones (Zhang, Lu, et al., 2021). Regarding this, in addition to supplementing common nutrients such as calcium (Ca), vitamin D (Vit D), phosphorus (P), protein, and other nutrients that are beneficial to bone, the regular and quantitative supplementation of folic acid for middle‐aged and older individuals is also of vital significance (Schisterman et al., 2020; Zhang, Li, et al., 2021). Folic acid, as a member of the B vitamin family, participates in the synthesis and metabolism of purines, thymine, amino acids, hemoglobin, and methyl compounds in the host (Rydlewicz et al., 2002), while the deficiency of folic acid may result in diseases such as megaloblastic anemia, leukopenia, and hyperhomocysteinemia, among which hyperhomocysteinemia has also been verified to be a crucial factor contributing to the development of atherosclerosis and osteoporosis (Stone et al., 2017). Of note, several studies have reported a negative link between homocysteine level and vitamin B12 (Vit B12) and folic acid levels in the host (Mehrpour et al., 2021; Wu et al., 2022), and the enhancement of homocysteine level in middle‐aged and older people has also been widely recognized as one of the risk factors for osteoporosis (Clements et al., 2022; Enneman et al., 2015). Taken together with the above findings, supplementing folic acid in daily diets is expected to prevent osteoporosis and subsequent osteoporotic fractures in middle‐aged and older people by improving the homocysteine level in the host.

Dietary folic acid supplementation might contribute to maintaining bone mass in the middle‐aged and elderly populations and prevent the occurrence and progression of osteoporosis (Stone et al., 2017). However, there are still few studies focusing on the significance of dietary intake and osteoporosis at the current stage, and there is also a lack of large‐scale population‐based studies to investigate the link between dietary folic acid intake and the risk of osteoporosis in middle‐aged and older people. Moreover, the dietary intake of folic acid is still relatively scarce in some countries and regions where diets are mainly characterized by cereals, and the digestive and absorption functions of middle‐aged and older people gradually deteriorate with the growth of age, which poses certain obstacles to the intake of dietary nutrients (Monteagudo et al., 2013; Steluti et al., 2019). Thus, it is crucial to analyze and recognize the link between dietary folic acid intake and the risk of osteoporosis among middle‐aged and older people. Herein, based on NHANES 2007–2010, this study aims to investigate the above correlation and further determine the cutoff value of daily dietary folic acid intake, thereby providing certain references for the prevention and treatment of osteoporosis in middle‐aged and older people from the perspective of dietary intake.

## 2 METHODS

### 2.1 Data provenance and research population

As a nationwide population‐based survey research program established by Centers for Disease Control and Prevention (CDC), NHANES is committed to noticing the health and nutritional status of non‐institutionalized adults and children in the US (Zhang et al., 2023). This research program has surveyed a nationally representative sample of approximately 5000 people annually, with the interviews covering demographic, socio‐economic, dietary, laboratory, and other health‐related matters. The examination section includes medical, dental, and physiological measurements, as well as laboratory tests managed by trained medical personnel (Hong et al., 2022). The survey findings might be applied to ascertain the prevalence and risk factors of the main diseases and then provide extensive information support for the formulation of nutrition and health policies (Nigra et al., 2017). NHANES conducts surveys every 2 years, and 2 years are defined as a study period, and the overall data are utterly de‐identified and publicly obtainable to researchers on the page of www.cdc.gov/nchs/nhanes. More significantly, this current study is performed according to the principles of the Declaration of Helsinki and is also a simply exempt study involving secondary processing data, which is approved by the Research Ethics Review Board of the National Center for Health Statistics (NCHS). The written informed consent of participants included in the NHANES is also obtained (Chen et al., 2019).

**FIGURE 1:**
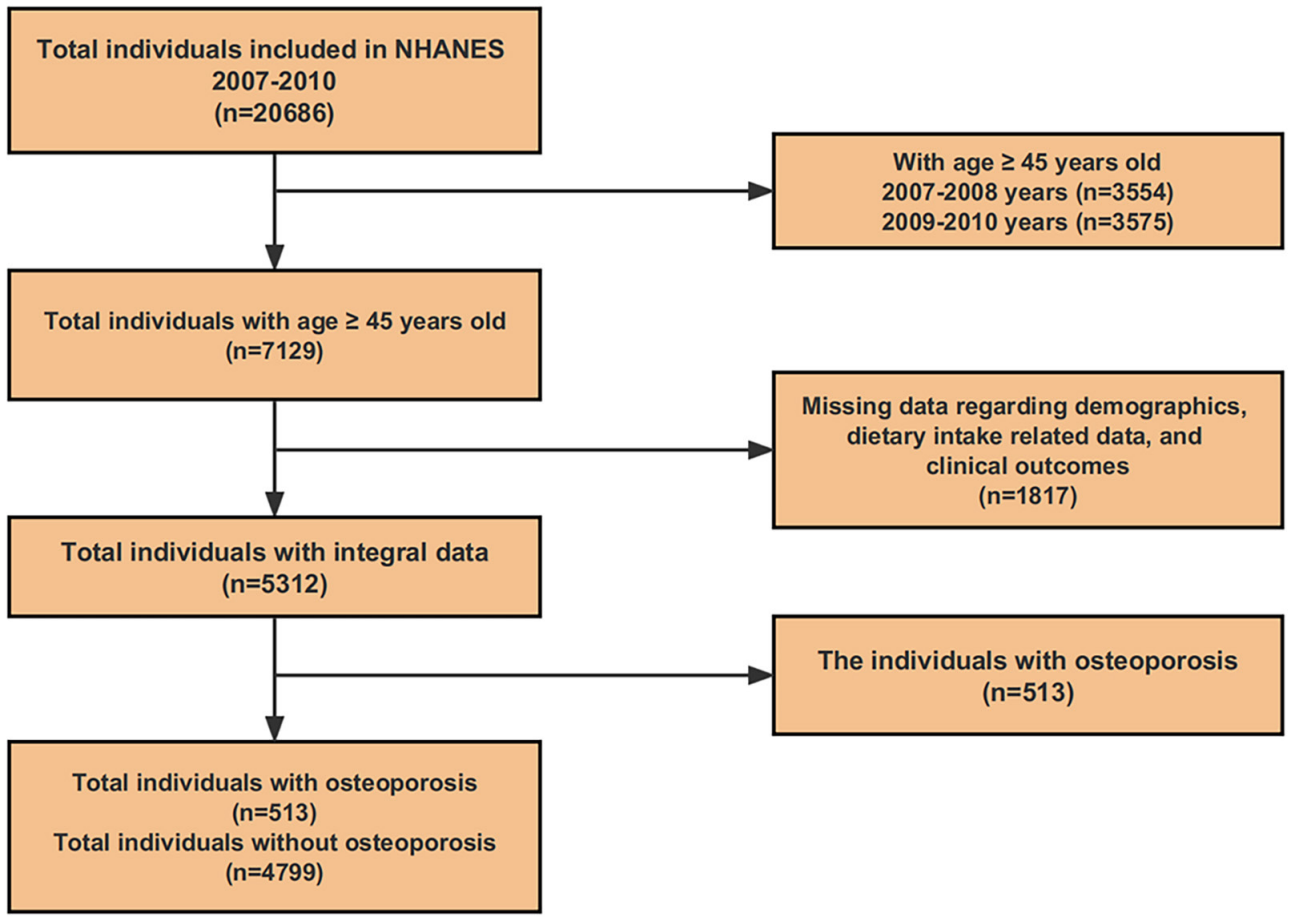
Flow chart screening based on NHANES 2007–2010.

As exhibited in Figure 1, a total of 20,686 people from NHANES 2007–2010 (2007–2008 and 2009–2010 years) are screened and included in this current study, and the research object is defined as the individuals aged greater than or equal to 45 years old (*n* = 7129). Subsequently, a total of 1817 people with absent data regarding demographic characteristics, dietary intake related data, and clinical consequences are eliminated, and a total of 5312 people with integral data are ultimately enrolled in the next evaluation.

### 2.2 Demographics

As a continuous cross‐sectional survey conducted over the years by the CDC, NHANES gathers data on the prevalence of chronic diseases and related indicators in the population. It is also able to understand the risk factors of various diseases, that is, the individual's lifestyle, constitution, genetics, and environmental factors that might enhance the risk of diseases, including smoking, alcohol consumption, sexual behavior, exercise, dietary intake, and other parameters (Jayanama et al., 2022). Moreover, the demographically relevant variables of NHANES 2007–2010, including age, race, gender, education level, body mass index (BMI), smoking, ratio of family income to poverty, drinking alcohol, and common comorbidities (such as hypertension and diabetes), are included in the subsequent analysis.

### 2.3 Identification of individuals with osteoporosis

In NHANES 2007–2010, bone mineral density (BMD) of the spine and femur among the included population was analyzed by means of dual‐energy X‐ray absorptiometry (DXA), with a diagnostic standard of osteoporosis as a T‐score lower than −2.5 standard deviation (SD) (Narayanasamy et al., 2022). Based on this diagnostic standard, the above enrolled individuals (*n* = 5312) are then separated into the osteoporosis group (*n* = 513) and the non‐osteoporosis group (*n* = 4799).

### 2.4 Evaluation of dietary intake‐related data

As a branch of data collection in NHANES 2007–2010, a research survey of “What We Eat in America” (WWEIA) is promoted by collaboration between two departments, including the US Department of Agriculture (USDA) and the US Department of Health and Human Services (USDHHS). Specifically, USDA is mainly committed to screening, collecting, and identifying dietary intake‐related data, and USDHHS is mainly committed to performing survey overviews and gathering baseline information (Archer et al., 2015). Therein, during a whole 24‐h period, the dietary intake‐related data of enrolled individuals is recorded in two recall interviews by the trained dietitians in the mobile examination centers (MECs) (Kirkpatrick et al., 2022), and the specific gathered dietary intake‐related indicators include total energy, carbohydrates, folic acid, total fat, P, zinc, choline, protein, Ca, iron, Vit B6, fiber, Vit B12, cholesterol, and so on. The Food and Nutrient Database for Dietary Studies 2.0 (FNDD 2.0) of the USDA is then applied to analyze the calculations of dietary intake‐related data (Shelton et al., 2021). Moreover, considering the potential impact of total energy intake on various kinds of dietary nutrients, we have also adjusted for the total energy intake by means of the residual method and further standardized it to 2000 kcal (El Kinany et al., 2018).

### 2.5 Statistical data analysis

Demographics, dietary intake‐related data, and clinical outcomes information in the NHANES 2007–2010 are extracted by R 4.0.3 software (R Foundation, Vienna, Austria) and statistically analyzed using SPSS 23.0 software (SPSS Inc., Chicago, IL, USA). The data extraction and analysis processes follow the definition in the NHANES. Then, in accordance with the NHANES analysis guide, we consider NHANES‐related sample weights, and a *p* value less than .05 is regarded as statistically significant.

Next, the continuous variables are presented in the form of mean ± SD, median (interquartile range), or count (percentage), and categorical variables are presented in the form of percentage or frequency. Continuous variables are compared based on the Mann–Whitney *U* test for non‐normally distributed variables and Student's *t*‐test for normally distributed variables. Categorical variables are analyzed and compared by means of Chi‐square test.

Subsequently, multivariate logistic regression models are constructed to investigate the link between the risk of osteoporosis and dietary folic acid intake among middle‐aged and older people, which is expressed in the form of odds ratio (OR) and 95% confidence interval (CI). Based on this, we further apply the receiver operating characteristic (ROC) curve to identify the optimal cutoff value of daily dietary folic acid intake for indicating the risk of osteoporosis among middle‐aged and older people. In accordance with the daily dietary folic acid intake amount below or equal to and above optimal cutoff value, the research object is then divided into two independent groups, and the OR and 95% CI of the higher dietary folic acid intake group in contrast with the lower group are further analyzed. The criteria for selection of potential confounding variables mainly refers to risk factors related to osteoporosis in middle‐aged and elderly people, including age, gender, BMI, smoking, drinking alcohol, as well as dietary nutrients related to bone health, such as calcium, vitamin D, phosphorus, protein, and so on (Levis & Lagari, 2012; Zhang, Song, et al., 2024).

## 3 RESULTS

### 3.1 Demographics

The demographics of individuals with or without osteoporosis based on NHANES 2007–2010 are displayed in Table 1. Specifically, a total of 5312 people with a mean age of 62.4 ± 11.0 years old are involved in the ultimate assessment, and it is also observed that the individuals in the osteoporosis group (68.2 ± 9.9 years old) are older than those in the non‐osteoporosis group (61.8 ± 10.9 years old, *p* < .001). Besides, significant differences are identified in terms of gender (*p* < .001), race (*p* < .001), smoking (*p* = .029), drinking alcohol (*p* < .001), combined with hypertension (*p* = .001), and ratio of family income to poverty (*p* = .002), except for the terms of education level, combined with diabetes, and BMI (*p* all > .05).

**TABLE 1:** Demographics of individuals with or without osteoporosis based on NHANES.

	Total (*n* = 5312)	Osteoporosis group (*n* = 513)	Non‐osteoporosis group (*n* = 4799)	*p* value
Age, years	62.4 ± 11.0	68.2 ± 9.9	61.8 ± 10.9	<.001
Gender (*n*, %)				<.001
Male	2657 (50.0)	78 (15.2)	2579 (53.7)	
Female	2655 (50.0)	435 (84.8)	2220 (46.3)	
Race, *n* (%)				<.001
Mexican American	793 (14.9)	73 (14.2)	720 (15.0)	
Other Hispanic	488 (9.2)	46 (9.0)	442 (9.2)	
Non‐Hispanic White	2836 (53.4)	325 (63.4)	2511 (52.3)	
Non‐Hispanic Black	1013 (19.1)	52 (10.1)	961 (20.0)	
Other race	182 (3.4)	17 (3.3)	165 (3.5)	
Education level, *n* (%)				.167
Less than 9th grade	783 (14.7)	81 (15.8)	702 (14.6)	
9–11th grade	854 (16.1)	83 (16.2)	771 (16.1)	
High school grad or equivalent	1255 (23.6)	137 (26.7)	1118 (23.3)	
Some college	1348 (25.4)	127 (24.8)	1221 (25.4)	
College graduate or above	1072 (20.2)	85 (16.5)	987 (20.6)	
Smoking (*n*, %)				.029
Yes	2729 (51.4)	240 (46.8)	2489 (51.9)	
No	2583 (48.6)	273 (53.2)	2310 (48.1)	
Drinking alcohol (*n*, %)				<.001
Yes	3651 (68.7)	283 (55.2)	3368 (70.2)	
No	1661 (31.3)	230 (44.8)	1431 (29.8)	
Combined with hypertension (*n*, %)				.001
Yes	2716 (51.1)	297 (57.9)	2419 (50.4)	
No	2596 (48.9)	216 (42.1)	2380 (49.6)	
Combined with diabetes (*n*, %)				.527
Yes	1001 (18.8)	102 (19.9)	899 (18.7)	
No	4311 (81.2)	411 (80.1)	3900 (81.3)	
Ratio of family income to poverty	2.7 ± 1.6	2.5 ± 1.6	2.7 ± 1.6	.002
BMI, kg/m^2^	29.4 ± 6.5	28.3 ± 6.4	29.5 ± 6.4	.525

Abbreviation: BMI, body mass index.

### 3.2 Dietary intake‐related data

The dietary intake‐related data of individuals with or without osteoporosis based on NHANES 2007–2010 is exhibited in Table 2. Specifically, in order to minimize the potential influences of total energy intake on various kinds of dietary nutrients, we have adjusted for the total energy intake by means of the residual method. It is observed that the energy‐adjusted dietary nutrients (such as protein, carbohydrates, total fat, fiber, Ca, P, zinc, Vit B6, iron, Vit B12, and cholesterol) of the individuals in the osteoporosis group are generally lower than those in the non‐osteoporosis group (*p* all < .05). It is also noticed that the daily dietary folic acid intake of the individuals in the osteoporosis group (294.1 ± 247.4 μg/day) is significantly less than that of the non‐osteoporosis group (341.6 ± 313.1 μg/day, *p* < .001), which is worthy of further attention and analysis.

**TABLE 2:** Dietary intake‐related data of individuals with or without osteoporosis based on NHANES.

	Total (*n* = 5312)	Osteoporosis group (*n* = 513)	Non‐osteoporosis group (*n* = 4799)	*p* value
Total energy, kcal	1938.2 ± 910.0	1666.5 ± 698.7	1967.2 ± 925.1	<.001
Energy‐adjusted protein, g/day	75.8 ± 39.0	64.6 ± 29.0	77.0 ± 39.8	<.001
Energy‐adjusted carbohydrates, g/day	234.0 ± 111.8	210.1 ± 92.4	236.5 ± 113.3	<.001
Energy‐adjusted total fat, g/day	73.7 ± 43.7	62.6 ± 33.7	74.9 ± 44.5	<.001
Energy‐adjusted fiber, g/day	16.1 ± 9.9	14.8 ± 8.3	16.3 ± 10.0	<.001
Energy‐adjusted choline, mg/day	322.2 ± 195.1	273.1 ± 146.2	327.4 ± 198.9	<.001
Energy‐adjusted Ca, mg/day	867.9 ± 542.7	813.0 ± 499.6	873.7 ± 546.8	.008
Energy‐adjusted P, mg/day	1255.4 ± 618.0	1106.4 ± 497.0	1271.3 ± 627.5	<.001
Energy‐adjusted iron, mg/day	14.3 ± 8.2	13.0 ± 6.6	14.5 ± 8.3	<.001
Energy‐adjusted zinc, mg/day	11.1 ± 9.2	9.6 ± 5.3	11.3 ± 9.6	<.001
Energy‐adjusted Vit B6, mg/day	1.9 ± 1.2	1.6 ± 1.0	1.9 ± 1.3	.001
Energy‐adjusted Vit B12, μg/day	5.1 ± 3.1	4.4 ± 2.8	5.2 ± 3.3	.027
Energy‐adjusted cholesterol, mg/day	280.1 ± 237.5	231.2 ± 180.3	285.3 ± 242.3	<.001
Energy‐adjusted folic acid, μg/day	337.0 ± 307.7	294.1 ± 247.4	341.6 ± 313.1	<.001

Abbreviations: Ca, calcium; P, phosphorus; Vit B6, Vitamin B6; Vit B12, Vitamin B12.

### 3.3 Link between the risk of osteoporosis and dietary folic acid intake

By means of constructing the multivariate logistic regression models, we then assess the link between the risk of osteoporosis and dietary folic acid intake. In this regard, Table 3 exhibits the prevalence and exact sample of osteoporosis in each tertile category of dietary folic acid intake amount, as well as the OR and 95% CI for osteoporosis according to different dietary folic acid intake levels. The prevalence of osteoporosis in each tertile category of dietary folic acid intake amount exhibits a gradual descending tendency (10.3%, 8.7%, and 6.5%, respectively, *p* for trend < .001). Based on this, the lowest tertile category is used to act as a reference category, and a higher dietary folic acid intake amount is observed to be positively correlated with lower odds for the risk of osteoporosis in middle‐aged and older people. More significantly, it is also noticed that the above‐mentioned trend is still not changed in the detached univariate model (*p* = .007), as well as adjustments for parameters of BMI, education level, ratio of family income to poverty, and combined chronic diseases (Model 1, *p* = .018), covariates of age and gender based on Model 1 (Model 2, *p* = .020), covariates of drinking alcohol and smoking based on Model 2 (Model 3, *p* = .027), and covariates of daily dietary intake data based on Model 3 (Model 4, *p* = .033).

**TABLE 3:** Odds ratios and 95% confidence intervals for the risk of osteoporosis judging by the daily dietary folic acid intake levels.

Dietary folic acid intake levels	Osteoporosis/*n*	Prevalence/%	Univariate model	Model 1^a^	Model 2^b^	Model 3^c^	Model 4^d^
Total (*n* = 5312)							
Tertile 1 (≤400 μg/day)	375/3627	10.3	1 (Ref.)	1 (Ref.)	1 (Ref.)	1 (Ref.)	1 (Ref.)
Tertile 2 (>400, ≤800 μg/day)	112/1285	8.7	0.715 (0.459–1.113)	0.714 (0.459–1.112)	0.731 (0.470–1.138)	0.730 (0.469–1.137)	0.733 (0.471–1.141)
Tertile 3 (>800 μg/day)	26/400	6.5	0.570 (0.377–0.862)	0.592 (0.392–0.894)	0.603 (0.399–0.910)	0.610 (0.404–0.921)	0.616 (0.408–0.931)
*p* for trend		<.001	.007	.018	.020	.027	.033

^a^ Model 1 adjusted for the covariates of education level, BMI, ratio of family income to poverty, and combined chronic diseases.

^b^ Model 2 adjusted for the covariates of age and gender based on Model 1.

^c^ Model 3 adjusted for the covariates of smoking and drinking alcohol based on Model 2.

^d^ Model 4 adjusted for the covariates of daily dietary intake data (including nutrients related to bone health such as calcium, vitamin D, phosphorus, and protein) based on Model 3.

**FIGURE 2:**
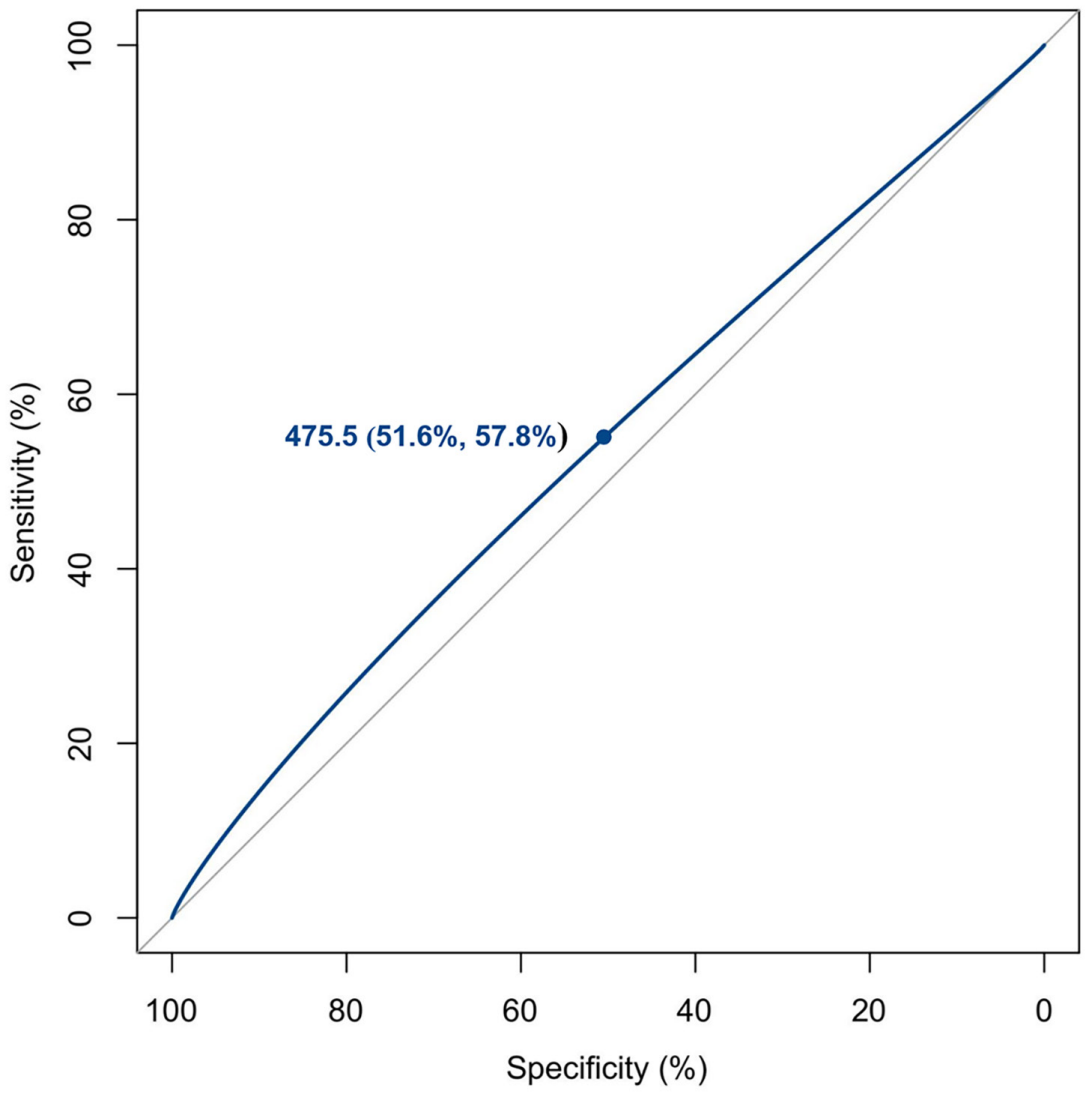
The dietary folic acid intake amount (475.5 μg/day) as an optimal cutoff value for revealing the risk of osteoporosis among middle‐aged and older people via the ROC curve, with 51.6% specificity and 57.8% sensitivity.

Based on the above‐mentioned results, Figure 2 reveals that the ROC curve is further used to investigate the dietary folic acid intake (475.5 μg/day) as an optimal cutoff value for revealing the risk of osteoporosis in middle‐aged and older individuals, with 51.6% specificity and 57.8% sensitivity. Furthermore, according to the dietary folic acid intake amount below or equal to and above optimal cutoff value (475.5 μg/day), research object is then divided into two independent groups. Next, the results of multivariate logistic regression models in Table 4 also indicate that individuals with dietary folic acid intake amount ≥475.5 μg/day are equipped with 0.636‐fold enhanced odds of osteoporosis in the detached univariate model, 0.645‐fold increased odds in Model 1, 0.774‐fold enhanced odds in Model 2, 0.663‐fold enhanced odds in Model 3, and 0.776‐fold enhanced odds in Model 4.

**TABLE 4:** Odds ratios and 95% confidence intervals for the risk of osteoporosis judging by the optimal cutoff value of daily dietary folic acid intake.

Daily dietary folic acid intake	Osteoporosis/*n*	Prevalence/%	Univariate model	Model 1^a^	Model 2^b^	Model 3^c^	Model 4^d^
Optimal cutoff value^e^							
Below	423/4019	10.5	1 (Ref.)	1 (Ref.)	1 (Ref.)	1 (Ref.)	1 (Ref.)
Equal and above	90/1293	7.0	0.636 (0.502–0.806)	0.645 (0.509–0.818)	0.774 (0.607–0.988)	0.663 (0.523–0.841)	0.776 (0.603–0.999)

^a^ Model 1 adjusted for the covariates of education level, BMI, ratio of family income to poverty, and combined chronic diseases.

^b^ Model 2 adjusted for the covariates of age and gender based on Model 1.

^c^ Model 3 adjusted for the covariates of smoking and drinking alcohol based on Model 2.

^d^ Model 4 adjusted for the covariates of daily dietary intake data (including nutrients related to bone health such as calcium, vitamin D, phosphorus, and protein) based on Model 3.

^e^ Optimal cutoff value of daily dietary folic acid intake is 475.5 μg.

## 4 DISCUSSION

With the enhancing aging of the global population, the prevalence of osteoporosis among middle‐aged and older people, as well as the combined disability and mortality rates, are also rapidly ascending (Zhang, Cao, Li, Chen, et al., 2022; Zhang, Cao, Li, Zhang, et al., 2022). In this context, healthy and balanced dietary intake is a pivotal cornerstone for maintaining bone and whole health among middle‐aged and older people (Fardellone et al., 2010), and it is also necessary to timely screen, identify, and discover the misconceptions and deficiencies in terms of the dietary intake among middle‐aged and older people. Herein, based on NHANES 2007–2010, this nationwide population‐based study reveals that a higher daily dietary folic acid intake has potential protective effects on osteoporosis among middle‐aged and elderly population, and it is also recommended to ensure that the daily dietary folic acid intake amount is greater than or equal to 475.5 μg to prevent the occurrence and development of osteoporosis.

This nationwide population‐based study mainly indicates that the individuals in the osteoporosis group are older than those in the non‐osteoporosis group (*p* < .001), and the daily dietary folic acid intake of individuals in the osteoporosis group is lower than that of the non‐osteoporosis group (*p* < .001), which suggests an underlying correlation between high daily dietary folic acid intake and low risk of osteoporosis among middle‐aged and older people. Regarding this, the above‐mentioned results are also consistent with several previous studies. A former study conducted by Keser et al. (2013) revealed that the application of folic acid and Vit B12 as dietary supplements to enhance the level of homocysteine could be conductive to middle‐aged and older women and positively modify the bone turnover markers among the above population. Rejnmark et al. (2008) showed in a population‐based cohort (including 1869 perimenopausal women) that a higher dietary folic acid intake, but not Vit B12 or B2, may exert the positive effects on BMD of perimenopausal women. Li et al. (2017) also indicated in a community‐based study that the BMD of the hip in middle‐aged and older patients with chronic stroke has decreased, and their dietary folic acid intake amount is also significantly lower than the recommended daily intake standard. Hence, this series of results emphasizes the potential advantages and necessity of dietary folic acid supplementation in maintaining bone health among middle‐aged and older populations, and deserves further attention and research.

Folic acid, as a vital nutrient for the human body, plays a pivotal role in bone growth and preventing bone loss. For one thing, oxidative stress, as an important risk factor for osteoporosis, has raised increasing attention from the research community, and folic acid, as a novel type of antioxidant, has also gradually become a research focus (Zhang et al., 2020). In this regard, several previous studies have suggested that folic acid may have direct impacts on osteoporosis, as it is required to participate in reducing oxidative stress, avoiding intracellular DNA damage, and preventing cell apoptosis (Mohammadi et al., 2012; Saito & Marumo, 2018; Tyagi et al., 2011). Meanwhile, low folic acid levels have also been reported to significantly decrease the thickness and number of trabeculae, and the supplementation of folic acid could rectify the coupling imbalance between the osteoblasts and osteoclasts in the host (Holstein et al., 2009; Kalimeri et al., 2020). Fan et al. (2012) observed through a study that the long‐term use of methotrexate (MTX) can damage the growth plate and metaphyseal bone cells of the body, while subsequent supplementation with folic acid might have protective effects. Specifically, the intake of chronic folic acid supplements can prevent the MTX‐induced apoptosis of chondrocytes and preserve the columnar arrangement and quantity of chondrocytes. Li et al. (Li et al., 2021) also noticed in a study that supplementation of dietary folic acid might improve the lipid metabolism, regulate the oxidative stress response, and activate the AMPK signaling pathway, thereby mitigating the high‐fat diet (HFD)‐induced osteoporosis. Thus, it can be recognized that folic acid plays a dominant role in the growth, progression, and homeostasis of bones, and a deficiency of folic acid might result in the descending bone formation and bone loss. Another potentially necessary factor for the occurrence of osteoporosis among middle‐aged and elderly populations is the subclinical deficiency of folic acid (Ratajczak et al., 2021). It is universally recognized that with the growth of age, middle‐aged and older people might be accompanied by tooth loss, decreased appetite, descending gastrointestinal absorption, and other weakening of body functions to a certain extent, thus resulting in inadequate intake of several daily dietary nutrients (Brazaitis et al., 2017; Sinikumpu et al., 2021). Hence, it is of great clinical significance to investigate the link between the risk of osteoporosis and dietary folic acid intake in the middle‐aged and elderly population and further explore relevant intervention measures to promote dietary folic acid intake based on this. Specifically, the following measures could more intuitively integrate dietary recommendations into current medical practices, including (1) Integrating nutritional interventions for dietary folic acid intake with healthcare systems to provide more sources of nutrient intake; (2) enhancing the investment and construction of nutritional departments in hospitals or health care units and further emphasizing the significance of nutritional support for maintaining resident health; and (3) strengthening the formulation and updating of clinical guidelines related to dietary nutrients, thereby guiding clinical practice more effectively (Saunders et al., 2023; Zhang, Wu, et al., 2024).

After identifying the correlation and significance between the risk of osteoporosis and dietary folic acid intake in the middle‐aged and elderly populations, we further verified the dietary folic acid intake amount (475.5 μg/day) as an optimal cutoff value to reveal the risk of osteoporosis in the middle‐aged and elderly populations. Regarding this, several studies have suggested that the daily dietary folic acid intake for most middle‐aged and older people is far below the recommended standards in the dietary nutrition guidelines for residents in multiple countries and regions around the world (Boilson et al., 2012; Olayiwola et al., 2014). Specifically, US dietary and nutritional guidelines have broadly recommended that middle‐aged and elderly people over 50 years old need to supplement approximately 400 μg of folic acid per day to maintain physical functions (Bailey et al., 2012). Although this has certain nutritional guidance significance, there is still a lack of specific evidence to indicate the dietary folic acid intake value required to maintain bone mass in middle‐aged and elderly people. From this, the value of this study lies in revealing that dietary folic acid intake should not be lower than 475.5 μg/day in middle‐aged and elderly people to avoid the occurrence of osteoporosis as much as possible. Meanwhile, the impacts of other potential factors (such as body size, dietary nutrient combinations, family dietary habits, cooking methods, and so on) on dietary folic acid intake in middle‐aged and elderly populations should also be closely monitored and focused on (Bailey & van Wijngaarden, 2015).

This nationwide population‐based study also has several shortcomings that need to be pointed out. First, different countries and regions around the world are equipped with different dietary characteristics, nutritional structures, lifestyles, and living habits (Fabiani et al., 2019). Based on the NHANES 2007–2010, this study mainly focuses on the middle‐aged and elderly population in the US, and the findings may not necessarily be generalizable to other ethnic or racial groups. In subsequent studies, it is still necessary to conduct large‐scale surveys of other ethnic groups and other age groups. Second, during the process of conducting studies on the correlation between chronic diseases and dietary nutrients, although we have considered and adjusted for partial potential confounding factors as far as possible, given the relatively complicated relationship between chronic diseases and dietary nutrients, some of the confounding factors are inevitably overlooked and cannot be ruled out. Third, it should be closely noted that the research based on NHANES is constrained by its self‐reporting nature. During the process of gathering baseline data and quantifying dietary intake‐related data from the individuals, there may inevitably be biases in the individuals' recall and self‐report. Fourth, as a cross‐sectional design, this current study focuses on a single point in time and cannot be used to analyze relationships over time or establish long‐term trends (Timpka et al., 2015). Fifth, precise control of dietary folic acid intake is particularly pivotal; excessive intake of folic acid can mask the symptoms of vitamin B12 deficiency (including anemia, fatigue, pigmentation, diarrhea, and so on), while long‐term vitamin B12 deficiency might cause irreversible neurological damage (Derin et al., 2016; Yang et al., 2020). Ultimately, the specificity and sensitivity percentages obtained in this study are not ideally conclusive, which is another major drawback that still needs to be pointed out. Despite this, NHANES contains a robust and nationwide dataset and has a stable and reliable data collection and analysis process. The secondary analysis of available progression data is also worthy of recognition, and the results of the analysis are worthy of confidence (Chen & Han, 2023; Shen et al., 2023).

## 5 CONCLUSION

Taken together, this nationwide population‐based study suggests that a higher daily dietary folic acid intake has potential protective effects on osteoporosis in middle‐aged and older people. Based on this, it is also recommended to ensure that their daily dietary folic acid intake amount is greater than or equal to 475.5 μg to avoid the occurrence and development of osteoporosis, although subsequent investigations in other countries and races around the world still need to be performed. Additionally, in response to the existing limitations, further research covering more countries, regions, and races is needed in the future to verify the relevance explored in this study and enhance its credibility.

## AUTHOR CONTRIBUTIONS

**Yuan‐Wei Zhang:** Conceptualization (equal); data curation (equal); formal analysis (equal); investigation (equal); methodology (equal). **Yan Hu:** Conceptualization (equal); data curation (equal); formal analysis (equal). **Si‐Cheng Wang:** Conceptualization (equal); data curation (equal); methodology (equal). **Zu‐Hao Li:** Data curation (equal); formal analysis (equal). **Gui‐Quan Cai:** Investigation (equal); methodology (equal). **Hao Shen:** Data curation (equal); formal analysis (equal). **Shi‐Hao Sheng:** Data curation (equal); investigation (equal). **Xiao Chen:** Conceptualization (equal); data curation (equal). **Wei‐Zong Weng:** Formal analysis (equal); methodology (equal). **Wen‐Cai Zhang:** Conceptualization (equal); methodology (equal). **Yuan Chen:** Conceptualization (equal); investigation (equal); supervision (equal); visualization (equal). **Jia‐Can Su:** Conceptualization (equal); data curation (equal); formal analysis (equal); funding acquisition (equal); investigation (equal); methodology (equal).

## FUNDING INFORMATION

The work was supported by grants from the General Program of China Postdoctoral Science Foundation (2023M742203), Integrated Project of Major Research Plan of National Natural Science Foundation of China (92249303), National Natural Science Foundation of China (82371603, 82230071), Shanghai Committee of Science and Technology Laboratory Animal Research Project (23141900600), Shanghai Hospital Development Center (SHDC2023CRT013), and Interdisciplinary of Medicine and Engineering Foundation of Shanghai JiaoTong University (YG2024QNA20, YG2024QNA21).

## CONFLICT OF INTEREST STATEMENT

The authors declare no conflicts of interest.

## ETHICS STATEMENT

The data from NHANES did not contain any identifiable or protected health information and was publicly available for public download by researchers. This current study was performed according to the principles of the Declaration of Helsinki and was also a simply exempt study involving secondary processing data, which was approved by the Research Ethics Review Board of NCHS.

## INFORMED CONSENT

Written informed consent was provided by the included participants in NHANES.

## Data Availability

The data that support the findings of this current study are available in NHANES at (https://wwwn.cdc.gov/nchs/nhanes/continuousnhanes/).

## References

[fsn34070-bib-0001] Archer, E. , Pavela, G. , & Lavie, C. J. (2015). The inadmissibility of what we eat in America and NHANES dietary data in nutrition and obesity research and the scientific formulation of national dietary guidelines. Mayo Clinic Proceedings, 90(7), 911–926. 10.1016/j.mayocp.2015.04.009 26071068 PMC4527547

[fsn34070-bib-0002] Bailey, R. L. , Fulgoni, V. L., 3rd , Keast, D. R. , & Dwyer, J. T. (2012). Examination of vitamin intakes among US adults by dietary supplement use. Journal of the Academy of Nutrition and Dietetics, 112(5), 657–663.e4. 10.1016/j.jand.2012.01.026 22709770 PMC3593649

[fsn34070-bib-0003] Bailey, R. L. , & van Wijngaarden, J. P. (2015). The role of B‐vitamins in bone health and disease in older adults. Current Osteoporosis Reports, 13(4), 256–261. 10.1007/s11914-015-0273-0 26017584

[fsn34070-bib-0004] Boilson, A. , Staines, A. , Kelleher, C. C. , Daly, L. , Shirley, I. , Shrivastava, A. , Bailey, S. W. , Alverson, P. B. , Ayling, J. E. , McDermott, A. , MacCooey, A. , Scott, J. M. , & Sweeney, M. R. (2012). Unmetabolized folic acid prevalence is widespread in the older Irish population despite the lack of a mandatory fortification program. The American Journal of Clinical Nutrition, 96(3), 613–621. 10.3945/ajcn.111.026633 22854405

[fsn34070-bib-0005] Brazaitis, M. , Paulauskas, H. , Eimantas, N. , Obelieniene, D. , Baranauskiene, N. , & Skurvydas, A. (2017). Heat transfer and loss by whole‐body hyperthermia during severe lower‐body heating are impaired in healthy older men. Experimental Gerontology, 96, 12–18. 10.1016/j.exger.2017.05.018 28554736

[fsn34070-bib-0006] Chen, C. , Ye, Y. , Zhang, Y. , Pan, X. F. , & Pan, A. (2019). Weight change across adulthood in relation to all cause and cause specific mortality: Prospective cohort study. BMJ, 367, l5584. 10.1136/bmj.l5584 31619383 PMC6812615

[fsn34070-bib-0007] Chen, J. , Zhang, H. , Wu, X. , Wang, F. , Wang, Y. , Gao, Q. , Liu, H. , Hu, Y. , Su, J. , & Jing, Y. (2021). PTHG2 reduces bone loss in ovariectomized mice by directing bone marrow mesenchymal stem cell fate. Stem Cells International, 2021, 8546739. 10.1155/2021/8546739 34976071 PMC8720025

[fsn34070-bib-0008] Chen, Y. , & Han, T. (2023). Cross‐sectional associations between healthy eating index and thyroid function in U.S. male adults, NHANES 2007‐2012. Food Science & Nutrition, 11(6), 2907–2914. 10.1002/fsn3.3270 37324875 PMC10261730

[fsn34070-bib-0009] Clements, M. , Heffernan, M. , Ward, M. , Hoey, L. , Doherty, L. C. , Hack Mendes, R. , Clarke, M. M. , Hughes, C. F. , Love, I. , Murphy, S. , McDermott, E. , Grehan, J. , McCann, A. , McAnena, L. B. , Strain, J. J. , Brennan, L. , & McNulty, H. (2022). A 2‐year randomized controlled trial with low‐dose B‐vitamin supplementation shows benefits on bone mineral density in adults with lower B12 status. Journal of Bone and Mineral Research, 37(12), 2443–2455. 10.1002/jbmr.4709 36128889 PMC10092614

[fsn34070-bib-0010] Derin, S. , Koseoglu, S. , Sahin, C. , & Sahan, M. (2016). Effect of vitamin B12 deficiency on olfactory function. International Forum of Allergy & Rhinology, 6(10), 1051–1055. 10.1002/alr.21790 27119316

[fsn34070-bib-0011] El Kinany, K. , Garcia‐Larsen, V. , Khalis, M. , Deoula, M. M. S. , Benslimane, A. , Ibrahim, A. , Benjelloun, M. C. , & El Rhazi, K. (2018). Adaptation and validation of a food frequency questionnaire (FFQ) to assess dietary intake in Moroccan adults. Nutrition Journal, 17(1), 61. 10.1186/s12937-018-0368-4 29895304 PMC5998554

[fsn34070-bib-0012] Enneman, A. W. , Swart, K. M. , van Wijngaarden, J. P. , van Dijk, S. C. , Ham, A. C. , Brouwer‐Brolsma, E. M. , van der Zwaluw, N. , Dhonukshe‐Rutten, R. A. , van der Cammen, T. , de Groot, L. C. , van Meurs, J. , Lips, P. , Uitterlinden, A. G. , Zillikens, M. C. , van Schoor, N. , & van der Velde, N. (2015). Effect of vitamin B12 and folic acid supplementation on bone mineral density and quantitative ultrasound parameters in older people with an elevated plasma homocysteine level: B‐PROOF, a randomized controlled trial. Calcified Tissue International, 96(5), 401–409. 10.1007/s00223-015-9968-6 25712255 PMC4415946

[fsn34070-bib-0013] Fabiani, R. , Naldini, G. , & Chiavarini, M. (2019). Dietary patterns in relation to low bone mineral density and fracture risk: A systematic review and meta‐analysis. Advances in Nutrition, 10(2), 219–236. 10.1093/advances/nmy073 30657847 PMC6416046

[fsn34070-bib-0014] Fan, C. M. , Foster, B. K. , Hui, S. K. , & Xian, C. J. (2012). Prevention of bone growth defects, increased bone resorption and marrow adiposity with folinic acid in rats receiving long‐term methotrexate. PLoS One, 7(10), e46915. 10.1371/journal.pone.0046915 23071661 PMC3465278

[fsn34070-bib-0015] Fardellone, P. , Cotté, F. E. , Roux, C. , Lespessailles, E. , Mercier, F. , & Gaudin, A. F. (2010). Calcium intake and the risk of osteoporosis and fractures in French women. Joint, Bone, Spine, 77(2), 154–158. 10.1016/j.jbspin.2009.08.007 20185352

[fsn34070-bib-0016] Farshbaf‐Khalili, A. , Ostadrahimi, A. , Heris, J. A. , Sarrafi, S. , & Mohammadisima, N. (2023). Dietary acid load is associated with primary osteoporosis in postmenopausal women aged 50‐65 years: A cross‐sectional study. Food Science & Nutrition, 11(2), 668–676. 10.1002/fsn3.3102 36789041 PMC9922108

[fsn34070-bib-0017] Guo, J. , Wang, F. , Hu, Y. , Luo, Y. , Wei, Y. , Xu, K. , Zhang, H. , Liu, H. , Bo, L. , Lv, S. , Sheng, S. , Zhuang, X. , Zhang, T. , Xu, C. , Chen, X. , & Su, J. (2023). Exosome‐based bone‐targeting drug delivery alleviates impaired osteoblastic bone formation and bone loss in inflammatory bowel diseases. Cell Reports Medicine, 4(1), 100881. 10.1016/j.xcrm.2022.100881 36603578 PMC9873828

[fsn34070-bib-0018] Holstein, J. H. , Herrmann, M. , Splett, C. , Herrmann, W. , Garcia, P. , Histing, T. , Graeber, S. , Ong, M. F. , Kurz, K. , Siebel, T. , Menger, M. D. , & Pohlemann, T. (2009). Low serum folate and vitamin B‐6 are associated with an altered cancellous bone structure in humans. The American Journal of Clinical Nutrition, 90(5), 1440–1445. 10.3945/ajcn.2009.28116 19759168

[fsn34070-bib-0019] Hong, Y. R. , Yadav, S. , Suk, R. , Lee, A. M. , Newsome, F. A. , Johnson‐Mann, C. N. , Cardel, M. I. , & Ross, K. M. (2022). Assessment of physical activity and healthy eating behaviors among US adults receiving bariatric surgery. JAMA Network Open, 5(6), e2217380. 10.1001/jamanetworkopen.2022.17380 35708688 PMC9204540

[fsn34070-bib-0020] Hu, Y. , Li, X. , Zhang, Q. , Gu, Z. , Luo, Y. , Guo, J. , Wang, X. , Jing, Y. , Chen, X. , & Su, J. (2021). Exosome‐guided bone targeted delivery of Antagomir‐188 as an anabolic therapy for bone loss. Bioactive Materials, 6(9), 2905–2913. 10.1016/j.bioactmat.2021.02.014 33718671 PMC7917458

[fsn34070-bib-0021] Hu, Y. , Li, X. , Zhi, X. , Cong, W. , Huang, B. , Chen, H. , Wang, Y. , Li, Y. , Wang, L. , Fang, C. , Guo, J. , Liu, Y. , Cui, J. , Cao, L. , Weng, W. , Zhou, Q. , Wang, S. , Chen, X. , & Su, J. (2021). RANKL from bone marrow adipose lineage cells promotes osteoclast formation and bone loss. EMBO Reports, 22(7), e52481. 10.15252/embr.202152481 34121311 PMC8406405

[fsn34070-bib-0022] Jayanama, K. , Theou, O. , Godin, J. , Mayo, A. , Cahill, L. , & Rockwood, K. (2022). Relationship of body mass index with frailty and all‐cause mortality among middle‐aged and older adults. BMC Medicine, 20(1), 404. 10.1186/s12916-022-02596-7 36280863 PMC9594976

[fsn34070-bib-0023] Kalimeri, M. , Leek, F. , Wang, N. X. , Koh, H. R. , Roy, N. C. , Cameron‐Smith, D. , Kruger, M. C. , Henry, C. J. , & Totman, J. J. (2020). Folate and vitamin B‐12 status is associated with bone mineral density and hip strength of postmenopausal Chinese‐Singaporean women. JBMR Plus, 4(10), e10399. 10.1002/jbm4.10399 33103028 PMC7574704

[fsn34070-bib-0024] Keser, I. , Ilich, J. Z. , Vrkić, N. , Giljević, Z. , & Colić Barić, I. (2013). Folic acid and vitamin B_12_ supplementation lowers plasma homocysteine but has no effect on serum bone turnover markers in elderly women: A randomized, double‐blind, placebo‐controlled trial. Nutrition Research, 33(3), 211–219. 10.1016/j.nutres.2013.01.002 23507227

[fsn34070-bib-0025] Kheiridoost, H. , Shakouri, S. K. , Shojaei‐Zarghani, S. , Dolatkhah, N. , & Farshbaf‐Khalili, A. (2022). Efficacy of nanomicelle curcumin, Nigella sativa oil, and their combination on bone turnover markers and their safety in postmenopausal women with primary osteoporosis and osteopenia: A triple‐blind randomized controlled trial. Food Science & Nutrition, 10(2), 515–524. 10.1002/fsn3.2674 35154688 PMC8825715

[fsn34070-bib-0026] Kirkpatrick, S. I. , Guenther, P. M. , Subar, A. F. , Krebs‐Smith, S. M. , Herrick, K. A. , Freedman, L. S. , & Dodd, K. W. (2022). Using short‐term dietary intake data to address research questions related to usual dietary intake among populations and subpopulations: Assumptions, statistical techniques, and considerations. Journal of the Academy of Nutrition and Dietetics, 122(7), 1246–1262. 10.1016/j.jand.2022.03.010 35283362

[fsn34070-bib-0027] Levis, S. , & Lagari, V. S. (2012). The role of diet in osteoporosis prevention and management. Current Osteoporosis Reports, 10(4), 296–302. 10.1007/s11914-012-0119-y 23001895

[fsn34070-bib-0028] Li, J. , Ho, W. T. P. , Liu, C. , Chow, S. K. , Ip, M. , Yu, J. , Wong, H. S. , Cheung, W. H. , Sung, J. J. Y. , & Wong, R. M. Y. (2021). The role of gut microbiota in bone homeostasis. Bone & Joint Research, 10(1), 51–59. 10.1302/2046-3758.101.Bjr-2020-0273.R1 33448869 PMC7845471

[fsn34070-bib-0029] Li, S. K. Y. , Wan, M. M. P. , Siu, F. P. L. , Chung, S. , & Pang, M. Y. C. (2017). Relationship between nutritional factors and hip bone density in individuals with chronic stroke. Calcified Tissue International, 101(3), 259–270. 10.1007/s00223-017-0276-1 28417148

[fsn34070-bib-0030] Li, Y. J. , Zhang, Y. W. , Cao, M. M. , Zhang, R. L. , Wu, M. T. , Rui, Y. F. , & Liu, N. F. (2024). The supplementation of Rothia as a potential preventive approach for bone loss in mice with ovariectomy‐induced osteoporosis. Food Science & Nutrition, 12(1), 340–353. 10.1002/fsn3.3747 38268892 PMC10804113

[fsn34070-bib-0031] Liu, H. , Li, M. , Zhang, T. , Liu, X. , Zhang, H. , Geng, Z. , & Su, J. (2022). Engineered bacterial extracellular vesicles for osteoporosis therapy. Chemical Engineering Journal, 450, 138309. 10.1016/j.cej.2022.138309

[fsn34070-bib-0032] Liu, H. , Zhang, H. , Wang, S. , Cui, J. , Weng, W. , Liu, X. , Tang, H. , Hu, Y. , Li, X. , Zhang, K. , Zhou, F. , Jing, Y. , & Su, J. (2023). Bone‐targeted bioengineered bacterial extracellular vesicles delivering siRNA to ameliorate osteoporosis. Composites Part B: Engineering, 255, 110610. 10.1016/j.compositesb.2023.110610

[fsn34070-bib-0033] Mehrpour, O. , Modi, M. , Mansouri, B. , Azadi, N. A. , Nakhaee, S. , Amirabadi, A. , Anaei‐Sarab, G. , Shirazi, F. M. , & Weiss, S. T. (2021). Comparison of vitamin B12, vitamin D, and folic acid blood levels in plumbism patients and controls in eastern Iran. Biological Trace Element Research, 199(1), 9–17. 10.1007/s12011-020-02119-6 32207029

[fsn34070-bib-0034] Miller, P. D. , Adachi, J. D. , Albergaria, B. H. , Cheung, A. M. , Chines, A. A. , Gielen, E. , Langdahl, B. L. , Miyauchi, A. , Oates, M. , Reid, I. R. , Santiago, N. R. , Vanderkelen, M. , Wang, Z. , & Yu, Z. (2022). Efficacy and safety of romosozumab among postmenopausal women with osteoporosis and mild‐to‐moderate chronic kidney disease. Journal of Bone and Mineral Research, 37(8), 1437–1445. 10.1002/jbmr.4563 35466448 PMC9544335

[fsn34070-bib-0035] Mills, E. S. , Hah, R. J. , Fresquez, Z. , Mertz, K. , Buser, Z. , Alluri, R. K. , & Anderson, P. A. (2022). Secondary fracture rate after vertebral osteoporotic compression fracture is decreased by anti‐osteoporotic medication but not increased by cement augmentation. The Journal of Bone and Joint Surgery. American Volume, 104(24), 2178–2185. 10.2106/jbjs.22.00469 36223482

[fsn34070-bib-0036] Mohammadi, A. , Omrani, L. , Omrani, L. R. , Kiani, F. , Eshraghian, A. , Azizi, Z. , & Omrani, G. R. (2012). Protective effect of folic acid on cyclosporine‐induced bone loss in rats. Transplant International, 25(1), 127–133. 10.1111/j.1432-2277.2011.01375.x 22039919

[fsn34070-bib-0037] Monteagudo, C. , Mariscal‐Arcas, M. , Palacin, A. , Lopez, M. , Lorenzo, M. L. , & Olea‐Serrano, F. (2013). Estimation of dietary folic acid intake in three generations of females in Southern Spain. Appetite, 67, 114–118. 10.1016/j.appet.2013.04.004 23587520

[fsn34070-bib-0038] Narayanasamy, M. , Bishop, S. , Sahota, O. , Paskins, Z. , Gittoes, N. , & Langley, T. (2022). Acceptability and engagement amongst patients on oral and intravenous bisphosphonates for the treatment of osteoporosis in older adults. Age and Ageing, 51(11), afac255. 10.1093/ageing/afac255 36413592

[fsn34070-bib-0039] Nigra, A. E. , Sanchez, T. R. , Nachman, K. E. , Harvey, D. , Chillrud, S. N. , Graziano, J. H. , & Navas‐Acien, A. (2017). The effect of the Environmental Protection Agency maximum contaminant level on arsenic exposure in the USA from 2003 to 2014: An analysis of the National Health and Nutrition Examination Survey (NHANES). The Lancet Public Health, 2(11), e513–e521. 10.1016/s2468-2667(17)30195-0 29250608 PMC5729579

[fsn34070-bib-0040] Olayiwola, I. O. , Fadupin, G. T. , Agbato, S. O. , & Soyewo, D. O. (2014). Serum micronutrient status and nutrient intake of elderly Yoruba people in a slum of Ibadan, Nigeria. Public Health Nutrition, 17(2), 455–461. 10.1017/s1368980012004971 23211101 PMC10282253

[fsn34070-bib-0041] Pinto, D. , Alshahrani, M. , Chapurlat, R. , Chevalley, T. , Dennison, E. , Camargos, B. M. , Papaioannou, A. , Silverman, S. , Kaux, J. F. , Lane, N. E. , Morales Torres, J. , Paccou, J. , Rizzoli, R. , Bruyere, O. , & Rehabilitation Working Group of IOF Committee of Scientific Advisors . (2022). The global approach to rehabilitation following an osteoporotic fragility fracture: A review of the rehabilitation working group of the International Osteoporosis Foundation (IOF) committee of scientific advisors. Osteoporosis International, 33(3), 527–540. 10.1007/s00198-021-06240-7 35048200

[fsn34070-bib-0042] Ratajczak, A. E. , Szymczak‐Tomczak, A. , Rychter, A. M. , Zawada, A. , Dobrowolska, A. , & Krela‐Kaźmierczak, I. (2021). Does folic acid protect patients with inflammatory bowel disease from complications? Nutrients, 13(11), 4036. 10.3390/nu13114036 34836291 PMC8618862

[fsn34070-bib-0043] Rejnmark, L. , Vestergaard, P. , Hermann, A. P. , Brot, C. , Eiken, P. , & Mosekilde, L. (2008). Dietary intake of folate, but not vitamin B2 or B12, is associated with increased bone mineral density 5 years after the menopause: Results from a 10‐year follow‐up study in early postmenopausal women. Calcified Tissue International, 82(1), 1–11. 10.1007/s00223-007-9087-0 18175033

[fsn34070-bib-0044] Rydlewicz, A. , Simpson, J. A. , Taylor, R. J. , Bond, C. M. , & Golden, M. H. (2002). The effect of folic acid supplementation on plasma homocysteine in an elderly population. QJM, 95(1), 27–35. 10.1093/qjmed/95.1.27 11834770

[fsn34070-bib-0045] Saito, M. , & Marumo, K. (2018). The effects of homocysteine on the skeleton. Current Osteoporosis Reports, 16(5), 554–560. 10.1007/s11914-018-0469-1 30116976

[fsn34070-bib-0046] Saunders, C. M. , Rehbinder, E. M. , Carlsen, K. C. L. , Jonassen, C. M. , LeBlanc, M. , Nordlund, B. , Skjerven, H. O. , Söderhäll, C. , Vettukattil, R. , & Carlsen, M. H. (2023). Feeding practices and dietary diversity in the first year of life: PreventADALL, a Scandinavian randomized controlled trial and birth cohort study. The Journal of Nutrition, 153(8), 2463–2471. 10.1016/j.tjnut.2023.06.015 37336319 PMC10447610

[fsn34070-bib-0047] Schisterman, E. F. , Sjaarda, L. A. , Clemons, T. , Carrell, D. T. , Perkins, N. J. , Johnstone, E. , Lamb, D. , Chaney, K. , van Voorhis, B. , Ryan, G. , Summers, K. , Hotaling, J. , Robins, J. , Mills, J. L. , Mendola, P. , Chen, Z. , DeVilbiss, E. , Peterson, C. M. , & Mumford, S. L. (2020). Effect of folic acid and zinc supplementation in men on semen quality and live birth among couples undergoing infertility treatment: A randomized clinical trial. JAMA, 323(1), 35–48. 10.1001/jama.2019.18714 31910279 PMC6990807

[fsn34070-bib-0048] Shelton, J. F. , Cameron, B. , Aslibekyan, S. , & Gentleman, R. (2021). Demographic, spatial and temporal dietary intake patterns among 526 774 23andMe research participants. Public Health Nutrition, 24(10), 2952–2963. 10.1017/s1368980020001251 32597744 PMC9884798

[fsn34070-bib-0049] Shen, X. , Yang, L. , Liu, Y. Y. , Jiang, L. , & Huang, J. F. (2023). Association between dietary niacin intake and cognitive function in the elderly: Evidence from NHANES 2011‐2014. Food Science & Nutrition, 11(8), 4651–4664. 10.1002/fsn3.3428 37576033 PMC10420858

[fsn34070-bib-0050] Sinikumpu, S. P. , Keränen, M. H. , Jokelainen, J. , Keinänen‐Kiukaanniemi, S. , & Huilaja, L. (2021). The association between chronic venous disease and measures of physical performance in older people: A population‐based study. BMC Geriatrics, 21(1), 556. 10.1186/s12877-021-02528-9 34649528 PMC8518156

[fsn34070-bib-0051] Steluti, J. , Reginaldo, C. , Selhub, J. , Paul, L. , Fisberg, R. M. , & Marchioni, D. M. (2019). Presence of circulating folic acid in plasma and its relation with dietary intake, vitamin B complex concentrations and genetic variants. European Journal of Nutrition, 58(8), 3069–3077. 10.1007/s00394-018-1852-5 30390106

[fsn34070-bib-0052] Stone, K. L. , Lui, L. Y. , Christen, W. G. , Troen, A. M. , Bauer, D. C. , Kado, D. , Schambach, C. , Cummings, S. R. , & Manson, J. E. (2017). Effect of combination folic acid, vitamin B_6_, and vitamin B_12_ supplementation on fracture risk in women: A randomized, controlled trial. Journal of Bone and Mineral Research, 32(12), 2331–2338. 10.1002/jbmr.3229 29244251 PMC5734110

[fsn34070-bib-0053] Timpka, T. , Janson, S. , Jacobsson, J. , Ekberg, J. , Dahlström, Ö. , Kowalski, J. , Bargoria, V. , Mountjoy, M. , & Svedin, C. G. (2015). Protocol design for large‐scale cross‐sectional studies of sexual abuse and associated factors in individual sports: Feasibility study in Swedish athletics. Journal of Sports Science and Medicine, 14(1), 179–187. 25729306 PMC4306771

[fsn34070-bib-0054] Tyagi, N. , Kandel, M. , Munjal, C. , Qipshidze, N. , Vacek, J. C. , Pushpakumar, S. B. , Metreveli, N. , & Tyagi, S. C. (2011). Homocysteine mediated decrease in bone blood flow and remodeling: Role of folic acid. Journal of Orthopaedic Research, 29(10), 1511–1516. 10.1002/jor.21415 21469179 PMC3583304

[fsn34070-bib-0055] Wang, L. , Yu, W. , Yin, X. , Cui, L. , Tang, S. , Jiang, N. , Cui, L. , Zhao, N. , Lin, Q. , Chen, L. , Lin, H. , Jin, X. , Dong, Z. , Ren, Z. , Hou, Z. , Zhang, Y. , Zhong, J. , Cai, S. , Liu, Y. , … Xia, W. (2021). Prevalence of osteoporosis and fracture in China: The China osteoporosis prevalence study. JAMA Network Open, 4(8), e2121106. 10.1001/jamanetworkopen.2021.21106 34398202 PMC8369359

[fsn34070-bib-0056] Warensjö, E. , Byberg, L. , Melhus, H. , Gedeborg, R. , Mallmin, H. , Wolk, A. , & Michaëlsson, K. (2011). Dietary calcium intake and risk of fracture and osteoporosis: Prospective longitudinal cohort study. BMJ, 342, d1473. 10.1136/bmj.d1473 21610048 PMC3101331

[fsn34070-bib-0057] Wu, Y. H. , Wu, Y. C. , Chang, J. Y. , Lee, Y. P. , Chiang, C. P. , & Sun, A. (2022). Significantly higher frequencies of macrocytosis, anemia, serum vitamin B12 and folic acid deficiencies, and hyperhomocysteinemia in male than in female atrophic glossitis patients. Journal of Dental Sciences, 17(3), 1371–1377. 10.1016/j.jds.2022.05.011 35784143 PMC9236945

[fsn34070-bib-0058] Yang, N. V. , Pannia, E. , Chatterjee, D. , Kubant, R. , Ho, M. , Hammoud, R. , Pausova, Z. , & Anderson, G. H. (2020). Gestational folic acid content alters the development and function of hypothalamic food intake regulating neurons in Wistar rat offspring post‐weaning. Nutritional Neuroscience, 23(2), 149–160. 10.1080/1028415x.2018.1479628 29848222

[fsn34070-bib-0059] Zhang, X. , Huang, Z. , Xie, Z. , Chen, Y. , Zheng, Z. , Wei, X. , Huang, B. , Shan, Z. , Liu, J. , Fan, S. , Chen, J. , & Zhao, F. (2020). Homocysteine induces oxidative stress and ferroptosis of nucleus pulposus via enhancing methylation of GPX4. Free Radical Biology & Medicine, 160, 552–565. 10.1016/j.freeradbiomed.2020.08.029 32896601

[fsn34070-bib-0060] Zhang, Y. W. , Cao, M. M. , Li, Y. J. , Chen, X. X. , Yu, Q. , & Rui, Y. F. (2022). A narrative review of the moderating effects and repercussion of exercise intervention on osteoporosis: Ingenious involvement of gut microbiota and its metabolites. Journal of Translational Medicine, 20(1), 490. 10.1186/s12967-022-03700-4 36303163 PMC9615371

[fsn34070-bib-0061] Zhang, Y. W. , Cao, M. M. , Li, Y. J. , Dai, G. C. , Lu, P. P. , Zhang, M. , Bai, L.‐Y. , Chen, X.‐X. , Shi, L. , Zhang, C. , & Rui, Y. F. (2022). Dietary protein intake in relation to the risk of osteoporosis in middle‐aged and older individuals: A cross‐sectional study. The Journal of Nutrition, Health & Aging, 26(3), 252–258. 10.1007/s12603-022-1748-1 35297468

[fsn34070-bib-0062] Zhang, Y. W. , Cao, M. M. , Li, Y. J. , Dai, G. C. , Lu, P. P. , Zhang, M. , Bai, L. Y. , Chen, X. X. , Zhang, C. , Shi, L. , & Rui, Y. F. (2022). The regulative effect and repercussion of probiotics and prebiotics on osteoporosis: Involvement of brain‐gut‐bone axis. Critical Reviews in Food Science and Nutrition, 63, 7510–7528. 10.1080/10408398.2022.2047005 35234534

[fsn34070-bib-0063] Zhang, Y. W. , Cao, M. M. , Li, Y. J. , Zhang, R. L. , Wu, M. T. , Yu, Q. , & Rui, Y. F. (2022). Fecal microbiota transplantation as a promising treatment option for osteoporosis. Journal of Bone and Mineral Metabolism, 40(6), 874–889. 10.1007/s00774-022-01375-x 36357745 PMC9649400

[fsn34070-bib-0064] Zhang, Y. W. , Li, Y. J. , Lu, P. P. , Dai, G. C. , Chen, X. X. , & Rui, Y. F. (2021). The modulatory effect and implication of gut microbiota on osteoporosis: From the perspective of “brain‐gut‐bone” axis. Food & Function, 12(13), 5703–5718. 10.1039/d0fo03468a 34048514

[fsn34070-bib-0065] Zhang, Y. W. , Lu, P. P. , Li, Y. J. , Dai, G. C. , Cao, M. M. , Xie, T. , Zhang, C. , Shi, L. , & Rui, Y. F. (2021). Low dietary choline intake is associated with the risk of osteoporosis in elderly individuals: A population‐based study. Food & Function, 12(14), 6442–6451. 10.1039/d1fo00825k 34076003

[fsn34070-bib-0066] Zhang, Y. W. , Lu, P. P. , Li, Y. J. , Wang, H. , Zhao, Y. K. , Chen, H. , & Rui, Y. F. (2023). Short report: Relationship between self‐reported sleep characteristics and falls‐associated fractures in elderly individuals: A population‐based study. Psychology, Health & Medicine, 28(4), 946–954. 10.1080/13548506.2022.2119482 36050909

[fsn34070-bib-0067] Zhang, Y. W. , Song, P. R. , Wang, S. C. , Liu, H. , Shi, Z. M. , & Su, J. C. (2024). Diets intervene osteoporosis via gut‐bone axis. Gut Microbes, 16(1), 2295432. 10.1080/19490976.2023.2295432 38174650 PMC10773645

[fsn34070-bib-0068] Zhang, Y. W. , Wu, Y. , Liu, X. F. , Chen, X. , & Su, J. C. (2024). Targeting the gut microbiota‐related metabolites for osteoporosis: The inextricable connection of gut‐bone axis. Ageing Research Reviews, 94, 102196. 10.1016/j.arr.2024.102196 38218463

[fsn34070-bib-0069] Zhou, R. X. , Zhang, Y. W. , Cao, M. M. , Liu, C. H. , Rui, Y. F. , & Li, Y. J. (2023). Linking the relation between gut microbiota and glucocorticoid‐induced osteoporosis. Journal of Bone and Mineral Metabolism, 41(2), 145–162. 10.1007/s00774-023-01415-0 36912997 PMC10010237

